# Case Report: Pathological Complete Response to Neoadjuvant Alectinib in a Patient With Resectable ALK-Positive Non-Small Cell Lung Cancer

**DOI:** 10.3389/fphar.2022.816683

**Published:** 2022-07-06

**Authors:** Yan Hu, Siying Ren, Ruoyao Wang, Wei Han, Peng Xiao, Li Wang, Fenglei Yu, Wenliang Liu

**Affiliations:** ^1^ Department of Thoracic Surgery, The Second Xiangya Hospital of Central South University, Hunan Key Laboratory of Early Diagnosis and Precision Treatment of Lung Cancer, The Second Xiangya Hospital of Central South University, Changsha, China; ^2^ Department of Respiratory and Critical Care Medicine, The Second Xiangya Hospital of Central South University, Research Unit of Respiratory Disease, Central South University, Hunan Diagnosis and Treatment Center of Respiratory Disease, Changsha, China; ^3^ Department of Cardiothoracic Surgery, The Third Xiangya Hospital of Central South University, Changsha, China

**Keywords:** pathological complete response, neoadjuvant therapy, alectinib, ALK, non-small cell lung cancer

## Abstract

**Background:** Alectinib, a highly selective inhibitor of ALK, is currently used in the first-line setting of untreated advanced ALK-positive NSCLC and in the second-line setting of crizotinib-resistant ALK-positive NSCLC. Despite promising efficacy and tolerability in the treatment of advanced ALK-positive NSCLC, the activity of alectinib as neoadjuvant therapy in resectable ALK-positive NSCLC remains to be investigated.

**Case presentation:** Herein, we report a case of a 58-year-old female patient presented to our hospital with hemoptysis for 1 month. Contrast-enhanced computerized tomography (CT) of the chest showed an approximately 4.2 × 3.4 cm mass in the right hilum with localized obstructive pneumonia in the right lower lobe and multiple enlarged lymph nodes in the right hilum and mediastinum. Serum oncological markers results showed elevated levels of CA19-9, CEA, CA125, and CA242. Bronchoscopic biopsy of the mass showed poorly differentiated pulmonary adenocarcinoma and immunohistochemical testing results confirmed ALK positivity. Neoadjuvant alectinib was given at a dosage of 600 mg twice per day for two cycles (56 days), achieving a partial response of the disease with 90% shrinkage of the mass at the subsequent whole-body positron emission tomography. Repeat serum oncological markers results showed that only CA125 was elevated, but lower than before therapy. A bilobectomy of the right middle and lower lobes and systemic lymphadectomy under video-assisted thoracoscopic approach was successfully performed 7 days after the last dose of alectinib. Postoperative pathology showed pathological complete response (pCR). The patient experienced an uneventful postoperative course and continued to receive alectinib and did not report any specific discomfort at her 8-month follow-up. Thoracoabdominal CT at 8 months postoperatively showed no recurrence and repeated examination of serum oncological markers were negative.

**Conclusion:** We report a case of resectable ALK-positive NSCLC treated with neoadjuvant aletinib achieving pCR. Our case highlights the feasibility of alectinib as neoadjuvant therapy for the treatment of resectable ALK-positive NSCLC. Undoubtedly, the safety and efficacy of this novel treatment modality needs to be explored in future large clinical trials.

## Introduction

Lung cancer is the leading cause of cancer-related deaths in China and worldwide (Sung et al., 2021). 733,000 new cases of non-small cell lung cancer (NSCLC) and approximately 610,000 deaths from non-small cell lung cancer occurred in China in 2015 (Chen et al., 2016). Non-small cell lung cancer accounts for about 85% of all lung cancers, of which 30–40% are “resectable”, including most stage I-IIIa and a small proportion of stage IIIb lung cancers (Goldstraw et al., 2016). For early-stage resectable lung cancer, radical surgical resection is the most important treatment. However, approximately 25–70% of resectable lung cancers will eventually recur after radical resection (Molina et al., 2008). Even with postoperative adjuvant therapy for appropriate patients, approximately 20–30% of stage I, 50% of stage II, and 60% of stage IIIa lung cancers still die within 5 years (Goldstraw et al., 2016).

In recent years, neoadjuvant therapy has increasingly become a valuable but controversial treatment modality (De Marinis et al., 2005). Neoadjuvant chemotherapy is effective in reducing tumor size and improving surgical resection rates, but has not shown survival advantage (NSCLC Meta-analysis Collaborative Group, 2014). Considering that targeted therapy have shown excellent therapeutic promise in metastatic lung cancer, some scholars have started to try them as neoadjuvant therapy for earlier stage lung cancer (Zhang et al., 2021).

Rearrangement of the anaplastic lymphoma kinase (ALK) is one of the important driver mutations in NSCLC, accounting for approximately 2–7% of NSCLC (Takeuchi et al., 2008). They represent a specific subgroup of NSCLC patients that are typically younger and light or non-smokers (Rodig et al., 2009). Alectinib, a highly selective inhibitor of ALK, is currently used in the first-line setting of untreated advanced ALK-positive NSCLC and in the second-line setting of crizotinib-resistant ALK-positive NSCLC (Peters et al., 2017). Despite promising efficacy and tolerability in the treatment of advanced ALK-positive NSCLC, the activity of alectinib as neoadjuvant therapy in resectable ALK-positive NSCLC remains to be investigated. Herein, we report the first case of resectable ALK-positive NSCLC receiving alectinib as neoadjuvant therapy followed by radical surgical resection to achieve a pCR.

## Case Presentation

**FIGURE 1:**
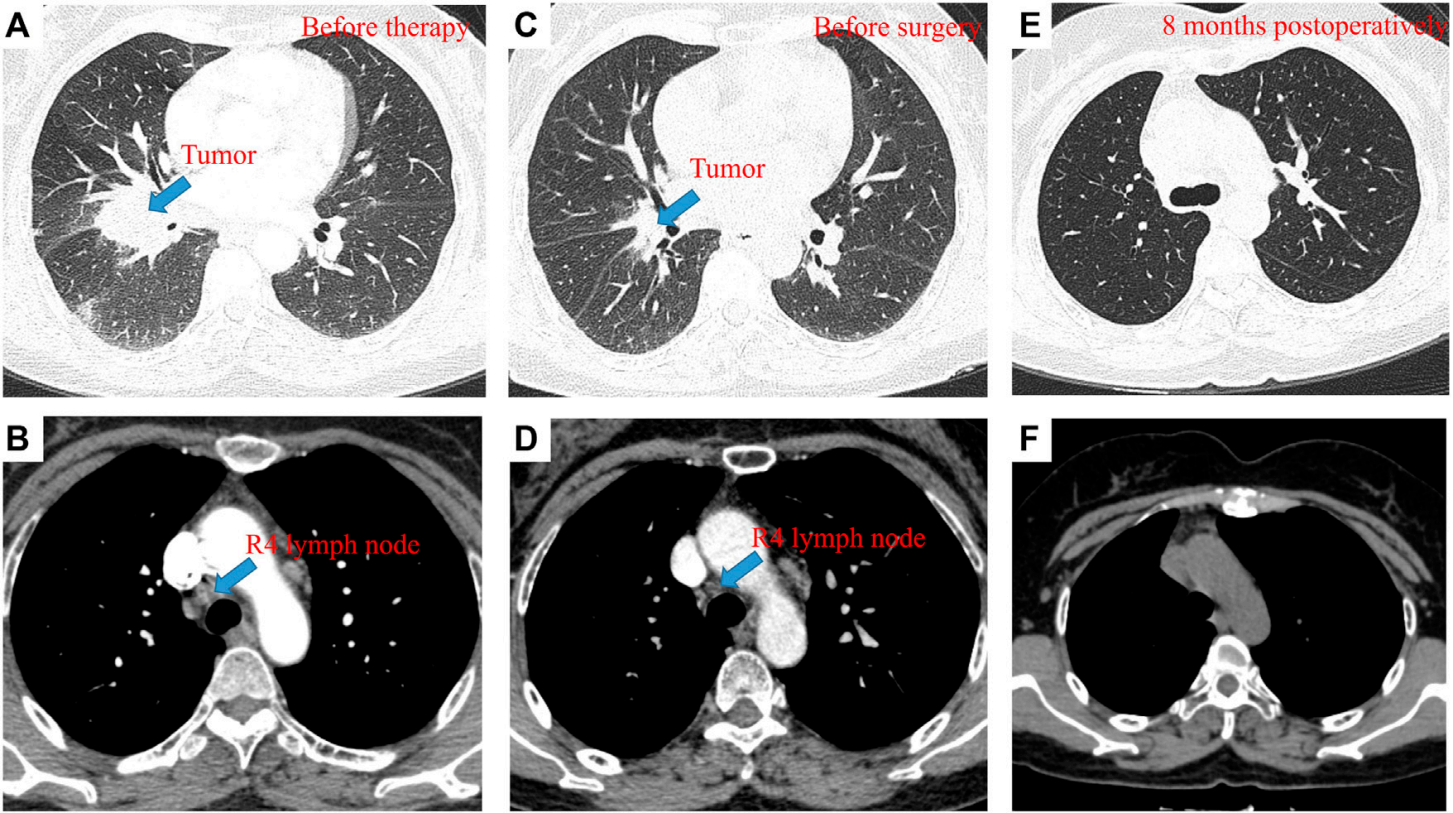
pCR to neoadjuvant alectinib in resectable ALK-positive NSCLC. Contrast-enhanced CT of the chest before neoadjuvant therapy showed an approximately 4.2 × 3.4 cm mass in the right hilum with localized obstructive pneumonia in the right lower lobe **(A)** and enlarged R4 lymph node **(B)**; Contrast-enhanced CT of the chest after neoadjuvant therapy showed an approximately 1.2 × 1.1 cm mass in the right hilum, with a tumor shrinkage of 90% **(C)** and 0.6 × 0.6 cm R4 lymph node, with a shrinkage of 50% **(D)**; Contrast-enhanced CT of the chest at the 8-month follow-up showed no local replase **(E,F)**.

**FIGURE 2:**
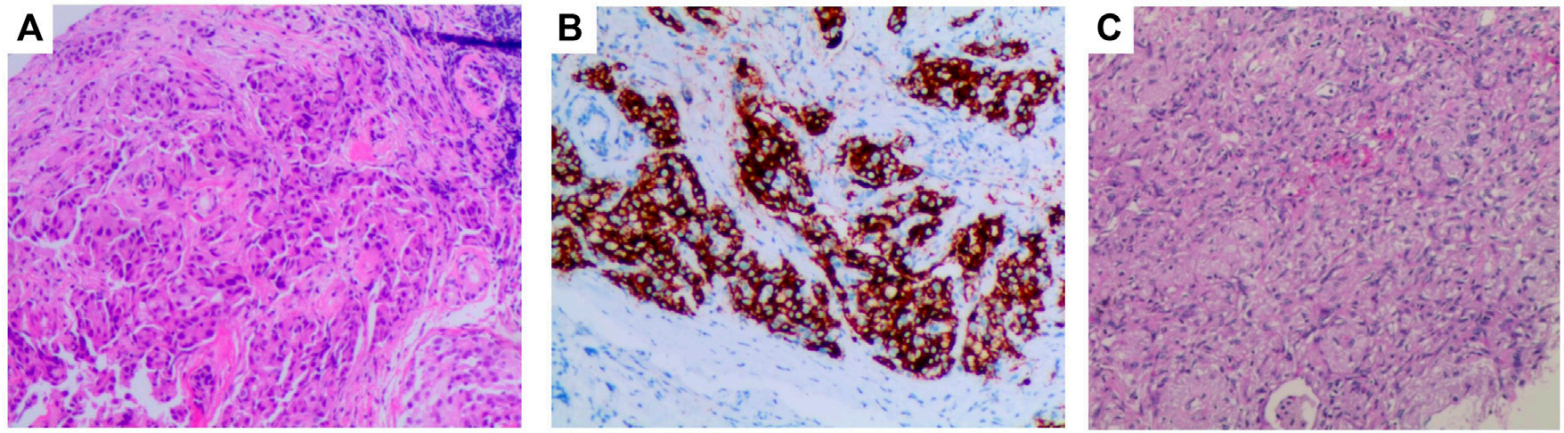
Pathologic findings of this patient. **(A)**, Bronchoscopic biopsy of the mass before treatment showed poorly differentiated pulmonary adenocarcinoma (H&E, × 100); **(B)**, Immunohistochemical testing results (D5F3 assay) confirmed strong positivity of ALK (× 100); **(C)**, Postoperative pathology showed pathological complete response, with a downstaging to ypT0N0M0 (H&E, × 100).

A 58-year-old female patient presented to our hospital in April 2021 with hemoptysis for 1 month. Contrast-enhanced computerized tomography (CT) of the chest showed an approximately 4.2 × 3.4 cm mass in the right hilum with localized obstructive pneumonia in the right lower lobe and multiple enlarged lymph nodes in the right hilum and mediastinum (Figure 1). Serum oncological markers results showed elevated levels of CA19-9 (61.04KU/L, reference value <35KU/L), CEA (37.63 ng/ml, reference value <5 ng/ml), CA125 (81.6KU/L, reference value <35KU/L) and CA242 (21.66KU/L, reference value <20KU/L). Routine blood biochemistry and pulmonary function tests showed no significant abnormalities. Fiberoptic bronchoscopy revealed significant mucosal infiltration and swelling in the bronchial opening of the right lower and middle lobes, and significant narrowing of the lumen of the lateral branch of the right middle lobe and the basal branch of the right lower lobe. Mucosal biopsy of the infiltrated lesion at the opening of the basal and dorsal segments of the right lower lobe showed poorly differentiated pulmonary adenocarcinoma with a Ki-67 of 35% (Figure 2). Immunohistochemical testing results (D5F3 assay, Ventana Medical Systems, Tucson, AZ) confirmed ALK positivity. A whole-body bone scan, contrast-enhanced MRI of the head, and contrast-enhanced CT of the abdomen were performed to rule out distant metastases.

**FIGURE 3:**
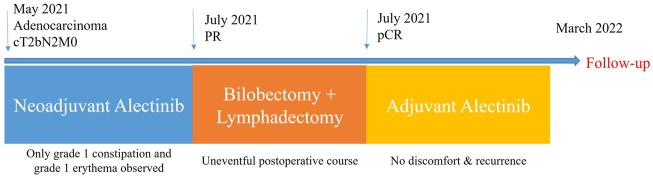
The timeline therapy administration from the episode of care. PR, partial response; pCR, pathological complete response.

After a multidisciplinary discussion, the patient was recommended to receive neoadjuvant alectinib therapy followed by surgical resection. Alectinib was given at a dosage of 600 mg twice per day for two cycles (56 days). Only grade 1 constipation and grade 1 erythema (located on the upper extremities) were observed during the neoadjuvant therapy. After the completion of two cycles of therapy, whole-body positron emission tomography was performed to assess the efficacy of neoadjuvant therapy. A partial response was achieved with neoadjuvant therapy, with 90% shrinkage of the mass (1.2 × 1.1 cm, SUVmax 3). 4R mediastinal lymph nodes showed mildly increased glucose metabolism with 50% shrinkage. Repeat serum oncological markers results showed that only CA125 (40.09 KU/L, reference value <35KU/L) was elevated, but lower than before therapy. A bilobectomy of the right middle and lower lobes and systemic lymphadectomy under video-assisted thoracoscopic approach was successfully performed 7 days after the last dose of alectinib. Postoperative pathology showed pCR, with a downstaging to ypT0N0M0. The patient experienced an uneventful postoperative course and was discharged on postoperative day 3. She continued to receive alectinib and did not report any specific discomfort at her 8-month follow-up. Thoracoabdominal CT at 8 months postoperatively showed no recurrence and repeated examination of serum oncological markers were negative, including CA125 dropping within normal range (19.34 KU/L, reference value <35KU/L). The timeline therapy administration from the episode of care was shown in Figure 3.

## Discussion

ALK is a transmembrane receptor tyrosine kinase that belongs to the insulin receptor superfamily. Since its discovery as a potential oncogenic driver in NSCLC in 2007 (Soda et al., 2007), ALK rearrangement can be detected in about 2–7% of NSCLC (Kwak et al., 2010). The most common ALK rearrangement in NSCLC is the juxtaposition of the 5′ end of the EML4 gene with the 3′ end of the ALK gene, giving rise to a new EML4-ALK fusion oncogene (Shaw and Solomon, 2011). EML4-ALK fusion protein induces dimerization of the intracellular kinase structural domain of ALK and activates classical PI3K/ALK/mTOR signaling, leading to cancer progression (Lovly et al., 2011).

ALK inhibitors have become the first-line treatment for advanced or metastatic ALK-positive NSCLC. Compared with chemotherapy, crizotinib treatment showed its superiority in patients with untreated advanced ALK-positive NSCLC regarding progression-free survival, objective response rate, cancer-related symptom relief, and quality of life improvement (Solomon et al., 2014). However, crizotinib has its limitation of rapidly developing resistance within 1–2 years of treatment (Friboulet et al., 2014). Alectinib, a second-generation ALK inhibitor, has proven to be effective against many of the major forms of crizotini-resistant ALK-positive NSCLC (Gadgeel et al., 2014). In addition, it showed superior systemic and central nervous system (CNS) efficacy and lower toxicity in untreated advanced ALK-positive NSCLC compared with crizotinib (Hida et al., 2017).

Neoadjuvant therapy, aimed at tumor downstaging and improving prognosis, is an emerging area of research. Most neoadjuvant targeted therapy studies are currently limited to patients with EGFR-mutated NSCLC. Although clinical trials assessing the efficacy of adjuvant alectinib therapy in resectable ALK-positive NSCLC are currently undergoing, evidence of alectinib in the neoadjuvant setting are lacking (Reyes and Reguart, 2021). Zhang et al. (2019) first reported 11 cases of pathologically confirmed N2 ALK-positive NSCLC treated with neoadjuvant crizotinib followed by surgery, 10 of which achieved R0 resection and 2 achieved pCR. Recently, Bing reported a case of stage IIIb crizotinib-resistant ALK-positive NSCLC that achieved pCR after receiving neoadjuvant ceritinib (Bing et al., 2021). Until now, only three cases have been reported regarding on the treatment of neoadjuvant alectinib in ALK-positive NSCLC. Zhang et al. (2020) reported a case of a stage cIIIb ALK-positive NSCLC patient who underwent two cycles of neoadjuvant alectinib therapy followed by radical surgical resection, with TNM stage downgraded to stage Ib. Yue et al. presented a case of cIIIa ALK-positive NSCLC patient who received one cycle of neoadjuvant alectinib therapy followed by surgery, with the tumor shrinkage of 42.2% and residual viable tumor cells of 15%. Leonetti et al. (2021) launched a phase II multicenter study to evaluate the efficacy and safety of neoadjuvant alectinib in resectable ALK-positive NSCLC and reported a case of a stage IIIa ALK-positive NSCLC patient treated with two cycles of neoadjuvant alectinib followed by surgery with a major pathological response (MPR). To the best of our knowledge, this is the first case of a patient with resectable ALK-positive NSCLC who received alectinib as neoadjuvant therapy to achieve pCR. No serious adverse events occurred during the neoadjuvant therapy and there were no intraoperative or postoperative complications occurred. No disease relapse was observed at the postoperative 8-month follow-up. The clinicopathological features of the previously reported cases and our case are summarized in Table 1.

**TABLE 1:** Summary of all cases of ALK-positive patient receiving neoadjuvant alectinib therapy.

	Zhang et al.	Yue et al.	Leonetti et al.	Present case
Age/gender	46/male	51/male	62/male	58/female
Symptoms	Cough and hemoptysis	None	NA	Hemoptysis
Smoking status	Nonsmoker	Nonsmoker	Former smoker	Nonsmoker
Location	Left lower lobe	Right upper lobe	Left upper lobe	Right lower lobe
Tumor size (cm)	6.6	3.1	NA	4.2
Baseline cTNM	cIIIb (cT3N2M0)	cIIIa (cT2N2M0)	cIIIa (cT2aN2M0)	cIIIa (cT2bN2M0)
Cycles	Two	One	Two	Two
Radiologic response	PR	PR	PR	PR
Pathologic response	non-MPR	non-MPR	MPR	pCR
Adverse effects	Grade 1 constipation	None	None	Grade 1 constipation
				Grade 1 erythema
Follow-up	NA	Free of disease for 6 months	NA	Free of disease for 8 months

PR, partial response; MPR, major pathological response; pCR, pathological complete response; NA, not available.

## Conclusion

In summary, we report a case of resectable ALK-positive NSCLC treated with neoadjuvant aletinib achieving pCR. Our case highlights the feasibility of alectinib as neoadjuvant therapy for the treatment of resectable ALK-positive NSCLC. Undoubtedly, the safety and efficacy of this novel treatment modality needs to be explored in future large clinical trials.

## Data Availability

The original contributions presented in the study are included in the article/supplementary material, further inquiries can be directed to the corresponding author.

## References

[B1] Bing Z. Jia Z. Wang Y. Xue J. Cao L. Cao Z. (2021). Pathological Complete Response to Neoadjuvant Ceritinib of a Crizotinib-Resistant, Stage IIIB Non-small Cell Lung Cancer with ALK Rearrangement: A Case Report. Thorac. Cancer 12 (14), 2130–2133. 10.1111/1759-7714.14045 34105864 PMC8287007

[B2] Chen W. Zheng R. Baade P. D. Zhang S. Zeng H. Bray F. (2016). Cancer Statistics in China, 2015. CA Cancer J. Clin. 66 (2), 115–132. 10.3322/caac.21338 26808342

[B3] De Marinis F. Gebbia V. De Petris L. (2005). Neoadjuvant Chemotherapy for Stage IIIA-N2 Non-small Cell Lung Cancer. Ann. Oncol. 16 (Suppl. 4), iv116–122. 10.1093/annonc/mdi920 15923411

[B4] Friboulet L. Li N. Katayama R. Lee C. C. Gainor J. F. Crystal A. S. (2014). The ALK Inhibitor Ceritinib Overcomes Crizotinib Resistance in Non-small Cell Lung Cancer. Cancer Discov. 4 (6), 662–673. 10.1158/2159-8290.CD-13-0846 24675041 PMC4068971

[B5] Gadgeel S. M. Gandhi L. Riely G. J. Chiappori A. A. West H. L. Azada M. C. (2014). Safety and Activity of Alectinib against Systemic Disease and Brain Metastases in Patients with Crizotinib-Resistant ALK-Rearranged Non-small-cell Lung Cancer (AF-002JG): Results from the Dose-Finding Portion of a Phase 1/2 Study. Lancet Oncol. 15 (10), 1119–1128. 10.1016/S1470-2045(14)70362-6 25153538

[B6] Goldstraw P. Chansky K. Crowley J. Rami-Porta R. Asamura H. Eberhardt W. E. (2016). The IASLC Lung Cancer Staging Project: Proposals for Revision of the TNM Stage Groupings in the Forthcoming (Eighth) Edition of the TNM Classification for Lung Cancer. J. Thorac. Oncol. 11 (1), 39–51. 10.1016/j.jtho.2015.09.009 26762738

[B7] Hida T. Nokihara H. Kondo M. Kim Y. H. Azuma K. Seto T. (2017). Alectinib versus Crizotinib in Patients with ALK-Positive Non-small-cell Lung Cancer (J-ALEX): an Open-Label, Randomised Phase 3 Trial. Lancet 390 (10089), 29–39. 10.1016/S0140-6736(17)30565-2 28501140

[B8] Kwak E. L. Bang Y. J. Camidge D. R. Shaw A. T. Solomon B. Maki R. G. (2010). Anaplastic Lymphoma Kinase Inhibition in Non-small-cell Lung Cancer. N. Engl. J. Med. 363 (18), 1693–1703. 10.1056/NEJMoa1006448 20979469 PMC3014291

[B9] Leonetti A. Minari R. Boni L. Gnetti L. Verzè M. Ventura L. (2021). Phase II, Open-Label, Single-Arm, Multicenter Study to Assess the Activity and Safety of Alectinib as Neoadjuvant Treatment in Surgically Resectable Stage III ALK-Positive NSCLC: ALNEO Trial. Clin. Lung Cancer 22 (5), 473–477. 10.1016/j.cllc.2021.02.014 33762169

[B10] Lovly C. M. Heuckmann J. M. de Stanchina E. Chen H. Thomas R. K. Liang C. (2011). Insights into ALK-Driven Cancers Revealed through Development of Novel ALK Tyrosine Kinase Inhibitors. Cancer Res. 71 (14), 4920–4931. 10.1158/0008-5472.CAN-10-3879 21613408 PMC3138877

[B11] Molina J. R. Yang P. Cassivi S. D. Schild S. E. Adjei A. A. (2008). Non-small Cell Lung Cancer: Epidemiology, Risk Factors, Treatment, and Survivorship. Mayo Clin. Proc. 83 (5), 584–594. 10.4065/83.5.584 18452692 PMC2718421

[B12] NSCLC Meta-analysis Collaborative Group (2014). Preoperative Chemotherapy for Non-small-cell Lung Cancer: a Systematic Review and Meta-Analysis of Individual Participant Data. Lancet 383 (9928), 1561–1571. 10.1016/S0140-6736(13)62159-5 24576776 PMC4022989

[B13] Peters S. Camidge D. R. Shaw A. T. Gadgeel S. Ahn J. S. Kim D. W. (2017). Alectinib versus Crizotinib in Untreated ALK-Positive Non-small-cell Lung Cancer. N. Engl. J. Med. 377 (9), 829–838. 10.1056/NEJMoa1704795 28586279

[B14] Reyes R. Reguart N. (2021). Neoadjuvant Treatment of Stage IIIA-N2 in EGFR-Mutant/ALK-Rearranged Non-small Cell Lung Cancer. Transl. Lung Cancer Res. 10 (1), 607–621. 10.21037/tlcr-20-780 33569340 PMC7867758

[B15] Rodig S. J. Mino-Kenudson M. Dacic S. Yeap B. Y. Shaw A. Barletta J. A. (2009). Unique Clinicopathologic Features Characterize ALK-Rearranged Lung Adenocarcinoma in the Western Population. Clin. Cancer Res. 15 (16), 5216–5223. 10.1158/1078-0432.CCR-09-0802 19671850 PMC2865649

[B16] Shaw A. T. Solomon B. (2011). Targeting Anaplastic Lymphoma Kinase in Lung Cancer. Clin. Cancer Res. 17 (8), 2081–2086. 10.1158/1078-0432.CCR-10-1591 21288922

[B17] Soda M. Choi Y. L. Enomoto M. Takada S. Yamashita Y. Ishikawa S. (2007). Identification of the Transforming EML4-ALK Fusion Gene in Non-small-cell Lung Cancer. Nature 448 (7153), 561–566. 10.1038/nature05945 17625570

[B18] Solomon B. J. Mok T. Kim D. W. Wu Y. L. Nakagawa K. Mekhail T. (2014). First-line Crizotinib versus Chemotherapy in ALK-Positive Lung Cancer. N. Engl. J. Med. 371 (23), 2167–2177. 10.1056/NEJMoa1408440 25470694

[B19] Sung H. Ferlay J. Siegel R. L. Laversanne M. Soerjomataram I. Jemal A. (2021). Global Cancer Statistics 2020: GLOBOCAN Estimates of Incidence and Mortality Worldwide for 36 Cancers in 185 Countries. CA Cancer J. Clin. 71 (3), 209–249. 10.3322/caac.21660 33538338

[B20] Takeuchi K. Choi Y. L. Soda M. Inamura K. Togashi Y. Hatano S. (2008). Multiplex Reverse Transcription-PCR Screening for EML4-ALK Fusion Transcripts. Clin. Cancer Res. 14 (20), 6618–6624. 10.1158/1078-0432.CCR-08-1018 18927303

[B21] Zhang C. Li S. L. Nie Q. Dong S. Shao Y. Yang X. N. (2019). Neoadjuvant Crizotinib in Resectable Locally Advanced Non-small Cell Lung Cancer with ALK Rearrangement. J. Thorac. Oncol. 14 (4), 726–731. 10.1016/j.jtho.2018.10.161 30408570

[B22] Zhang C. Yan L. X. Jiang B. Y. Wu Y. L. Zhong W. Z. (2020). Feasibility and Safety of Neoadjuvant Alectinib in a Patient with ALK-Positive Locally Advanced NSCLC. J. Thorac. Oncol. 15 (6), e95–e9. 10.1016/j.jtho.2019.12.133 32471573

[B23] Zhang Y. Fu F. Hu H. Wang S. Li Y. Hu H. (2021). Gefitinib as Neoadjuvant Therapy for Resectable Stage II-IIIA Non-small Cell Lung Cancer: A Phase II Study. J. Thorac. Cardiovasc Surg. 161 (2), 434. e2. 10.1016/j.jtcvs.2020.02.131 32340810

